# Disease of the Pancreas—Diagnosis

**Published:** 1851-07

**Authors:** 


*Disease of the Pancreas—Diagnosis.* By Dr. Fearnside.—After detailing two cases of obscure abdominal disease, in which, after death, the pancreas was the organ chiefly affected, Dr. Fearnside offers the following remarks in diagnosis :—

The earliest phenomena in both cases were the gradual failure of the general health and faulty digestion. To this succeeded *deep-seated pain in the epigastrium*, aggravated a few hours after food ; but this he observes may be due to a concomitant irritation of the mucous membrane of the stomach. *Gastrorrhœa* was present in both cases, but as the pancreas was diseased to such an extent as to prevent secretion, he justly supposes this symptom to be due to vitiated action of the mucous membrane of the stomach.

In one case *ptyalism* was a prominent symptom, and has been noticed in other cases of pancreatic disease. The tongue was *pale and clean* in both cases; the appetite variable.

In one case, *abdominal pulsation* was present. This is a symptom much insisted upon by professor Siebold, when cöexisting with other signs. The urine did not assist the diagnosis. *Emaciation and anæmia* were marked symptoms. From a comparison of these symptoms the author concludes, that though none are in themselves pathognomonic, the reunion of several affords strong probability of pancreatic disease.—*Medical Gazette, December 6th,* 1850.

